# Supplementary material for: Dynamic Changes of Bacterial Communities and Microbial Association Networks in Ready-to-Eat Chicken Meat during Storage
Source: Foods. 2022 Nov 21;11(22):3733. doi: 10.3390/foods11223733 (PMC9689599; doi:10.3390/foods11223733)
Supplement: Supplementary file 1 [file foods-11-03733-s001.zip › foods-2029117-supplementary.pdf]

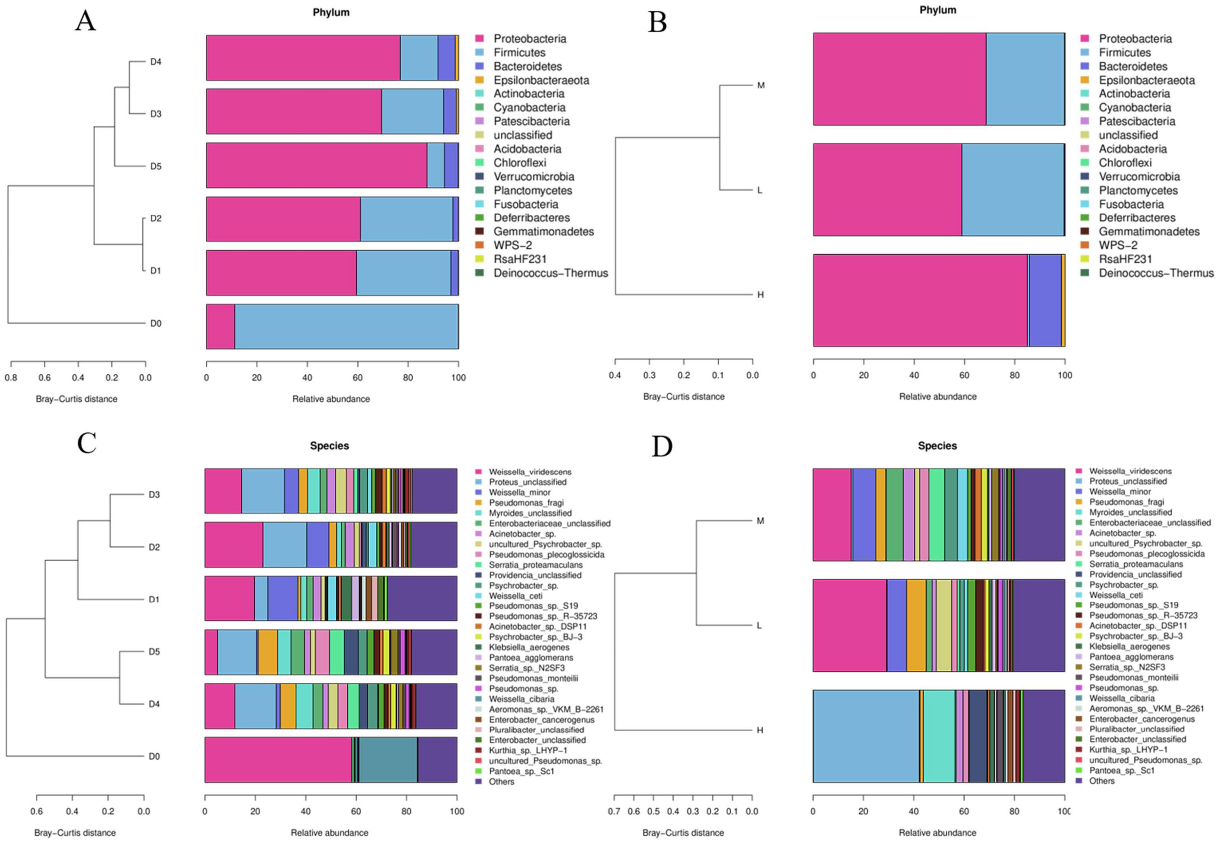

Figure S1. Cluster analysis in the RTE chicken meat samples based on (A, C) storage time and (B, D) temperature at the (A, B) phylum and (C, D) species level.

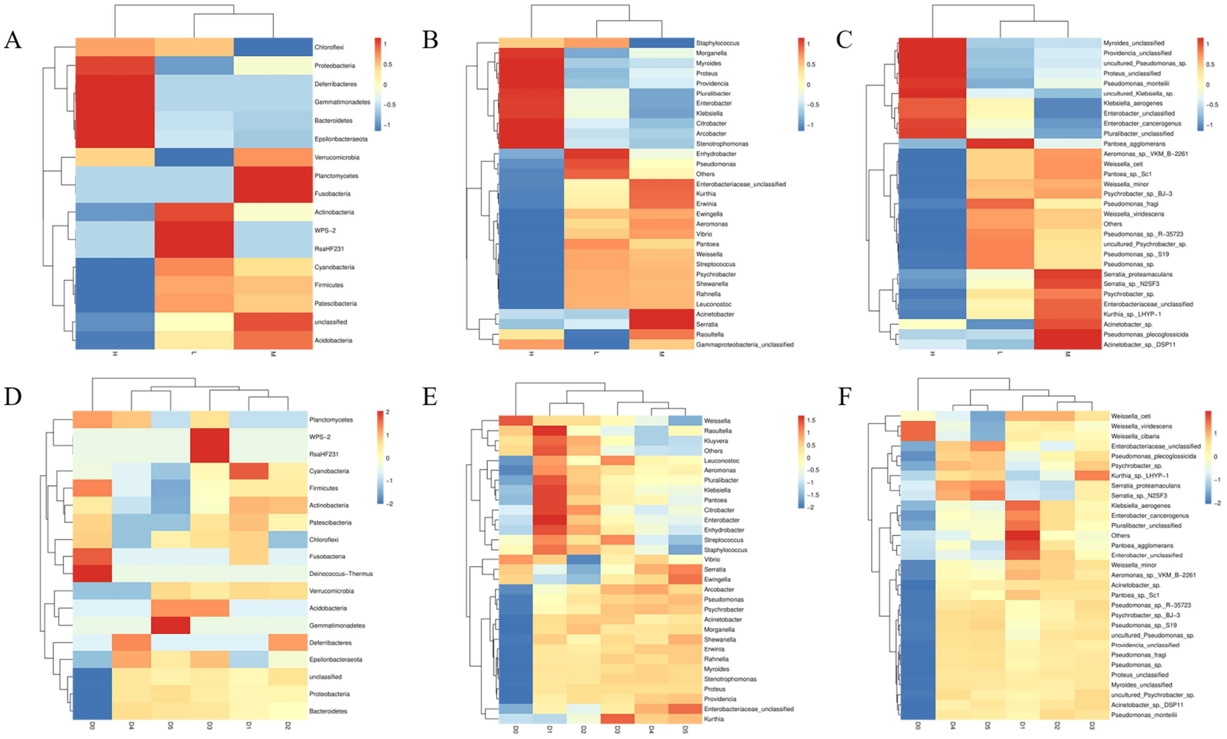

Figure S2. Heatmap analysis in the RTE chicken meat samples based on (A - C) storage temperature and (D - F) time at the (A, D) phylum, (B, E) genus and (C, F) species level.

Table S1. Species-level microbial interactions detected by SparCC in the bacterial communities of chicken meat samples stored at 4, 8 and 22 °C.

Temperature (°C)	Interaction	Pearson correlation†	Relation
4	*Weissella_minor ↔ Weissella_viridescens*	0.653	Copresence
	Pseudomonas_fragi ↔ Pseudomonas_sp._S19	0.6491	Copresence
	Pseudomonas_fragi ↔ Pseudomonas_sp.	0.5624	Copresence
	Pseudomonas_sp._R-35723 ↔ Pseudomonas_sp._S19	0.5401	Copresence
	Pseudomonas_fragi ↔ Pseudomonas_sp._R-35723	0.5241	Copresence
	Pseudomonas_sp. ↔ Pseudomonas_sp._S19	0.4964	Copresence
	Pseudomonas_sp. ↔ Pseudomonas_sp._R-35723	0.4567	Copresence
	Pseudomonas_fragi ↔ Pseudomonas_sp._55D	0.4556	Copresence
	Weissella_ceti ↔ Weissella_minor	0.4311	Copresence
	Pseudomonas_sp._55D ↔ Pseudomonas_sp._S19	0.4305	Copresence
	Psychrobacter_sp. ↔ Psychrobacter_sp._BJ-3	0.4186	Copresence
	Pseudomonas_sp._55D ↔ Weissella_viridescens	-0.4597	Mutual exclusion
	Pseudomonas_sp._R-35723 ↔ Weissella_minor	-0.4649	Mutual exclusion
	Pseudomonas_sp. ↔ Weissella_minor	-0.4682	Mutual exclusion
	Pseudomonas_sp ↔ Weissella_viridescens	-0.482	Mutual exclusion
	Pseudomonas_sp._R-35723 ↔ Weissella_viridescens	-0.5245	Mutual exclusion
	Pseudomonas_sp._S19 ↔ Weissella_minor	-0.5452	Mutual exclusion
	Pseudomonas_sp._S19 ↔ Weissella_viridescens	-0.5982	Mutual exclusion
	Pseudomonas_fragi ↔ Weissella_viridescens	-0.6072	Mutual exclusion
	Pseudomonas_fragi ↔ Weissella_minor	-0.6223	Mutual exclusion
8	*Weissella_minor ↔ Weissella_viridescens*	0.7217	Copresence
	Serratia_proteamaculans ↔ Serratia_sp._N2SF3	0.6608	Copresence
	Weissella_ceti ↔ Weissella_minor	0.6434	Copresence
	Enterobacteriaceae_unclassified ↔ Serratia_proteamaculans	0.6273	Copresence
	Pseudomonas_plecoglossicida ↔ Serratia_proteamaculans	0.6183	Copresence
	Psychrobacter_sp. ↔ Psychrobacter_sp._BJ-3	0.6125	Copresence
	Weissella_ceti ↔ Weissella_viridescens	0.586	Copresence
	Acinetobacter_sp. ↔ Acinetobacter_sp._DSP11	0.5672	Copresence
	Enterobacteriaceae_unclassified ↔ Serratia_sp._N2SF3	0.567	Copresence
	Pseudomonas_fragi ↔ Pseudomonas_sp._S19	0.5039	Copresence
	Pseudomonas_plecoglossicida ↔ Serratia_sp._N2SF3	0.4947	Copresence
	Enterobacteriaceae_unclassified ↔ Pseudomonas_plecoglossicida	0.4714	Copresence
	Pseudomonas_fragi ↔ Pseudomonas_sp._R-35723	0.454	Copresence
	Pseudomonas_fragi ↔ Pseudomonas_sp.	0.4321	Copresence
	Pseudomonas_fragi ↔ Psychrobacter_sp.	0.42	Copresence
	Acinetobacter_sp. ↔ Pseudomonas_plecoglossicida	0.4048	Copresence
	Pseudomonas_sp._R-35723 ↔ Pseudomonas_sp._S19	0.4035	Copresence
	Serratia_sp._N2SF3 ↔ Weissella_ceti	-0.4009	Mutual exclusion
	Pseudomonas_fragi ↔ Weissella_viridescens	-0.4098	Mutual exclusion
	Enterobacteriaceae_unclassified ↔ Weissella_minor	-0.4246	Mutual exclusion
	Serratia_proteamaculans ↔ Weissella_ceti	-0.425	Mutual exclusion
	Serratia_sp._N2SF3 ↔ Weissella_minor	-0.438	Mutual exclusion
	Enterobacteriaceae_unclassified ↔ Weissella_viridescens	-0.4794	Mutual exclusion
	Serratia_proteamaculans ↔ Weissella_minor	-0.4807	Mutual exclusion
	Serratia_sp._N2SF3 ↔ Weissella_viridescens	-0.5003	Mutual exclusion
	Serratia_proteamaculans ↔ Weissella_viridescens	-0.5295	Mutual exclusion
22	*Proteus_unclassified ↔ Providencia_unclassified*	0.4832	Copresence
	Klebsiella_aerogenes ↔ Pluralibacter_unclassified	0.4312	Copresence
	Enterobacter_unclassified ↔ Klebsiella_aerogenes	0.4211	Copresence
	Klebsiella_aerogenes ↔ Pantoea_agglomerans	0.4129	Copresence
	Proteus_unclassified ↔ Pseudomonas_plecoglossicida	0.4059	Copresence
	Klebsiella_aerogenes ↔ Providencia_unclassified	-0.4377	Mutual exclusion
	Enterobacter_unclassified ↔ Proteus_unclassified	-0.4402	Mutual exclusion
	Acinetobacter_sp. ↔ Providencia_unclassified	-0.4439	Mutual exclusion
	Pantoea_agglomerans ↔ Proteus_unclassified	-0.4812	Mutual exclusion
	Pluralibacter_unclassified ↔ Proteus_unclassified	-0.4866	Mutual exclusion
	Klebsiella_aerogenes ↔ Proteus_unclassified	-0.5782	Mutual exclusion

†The threshold values of Pearson correlation in the three constructed networks were >0.4 or <-0.4.
